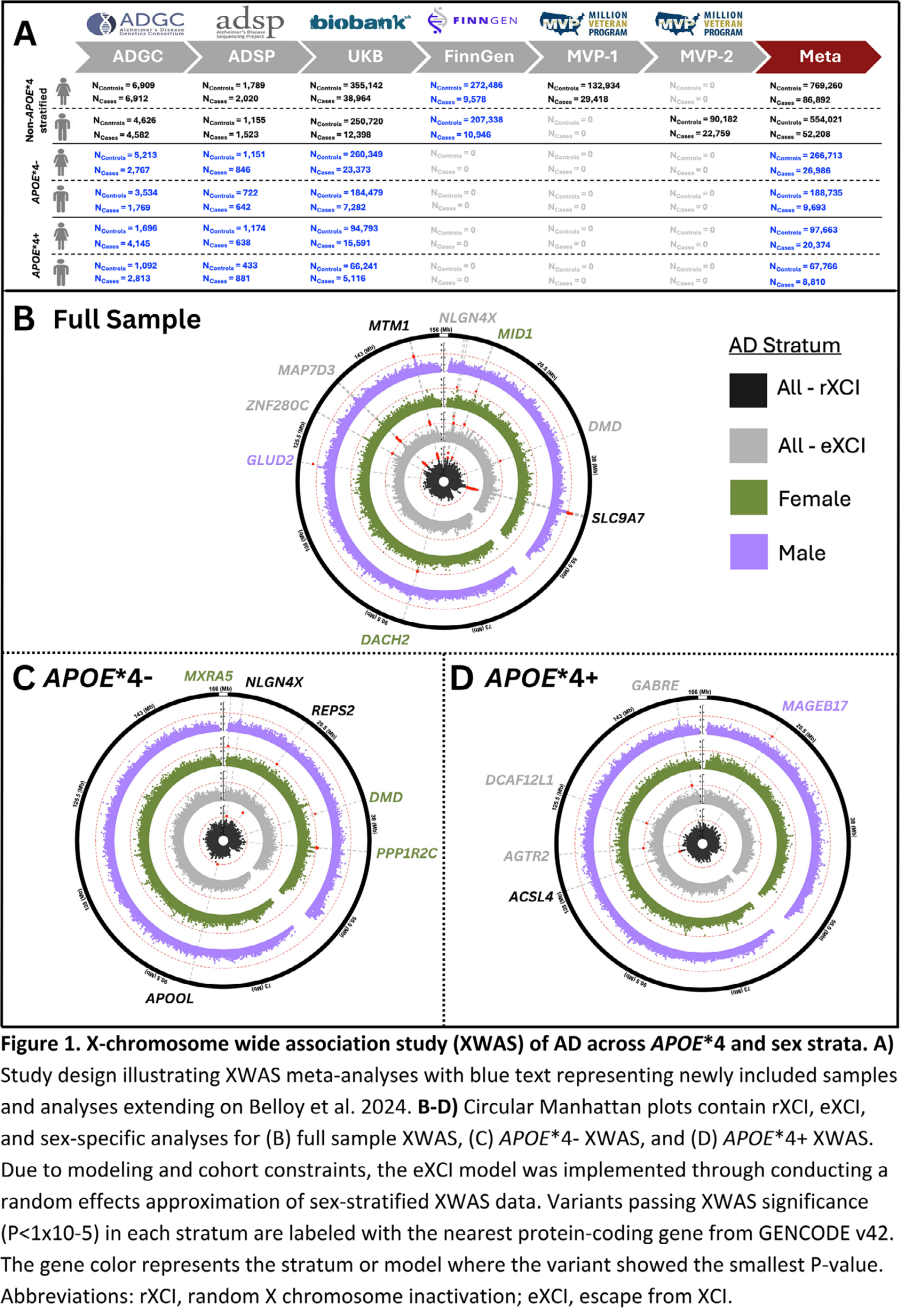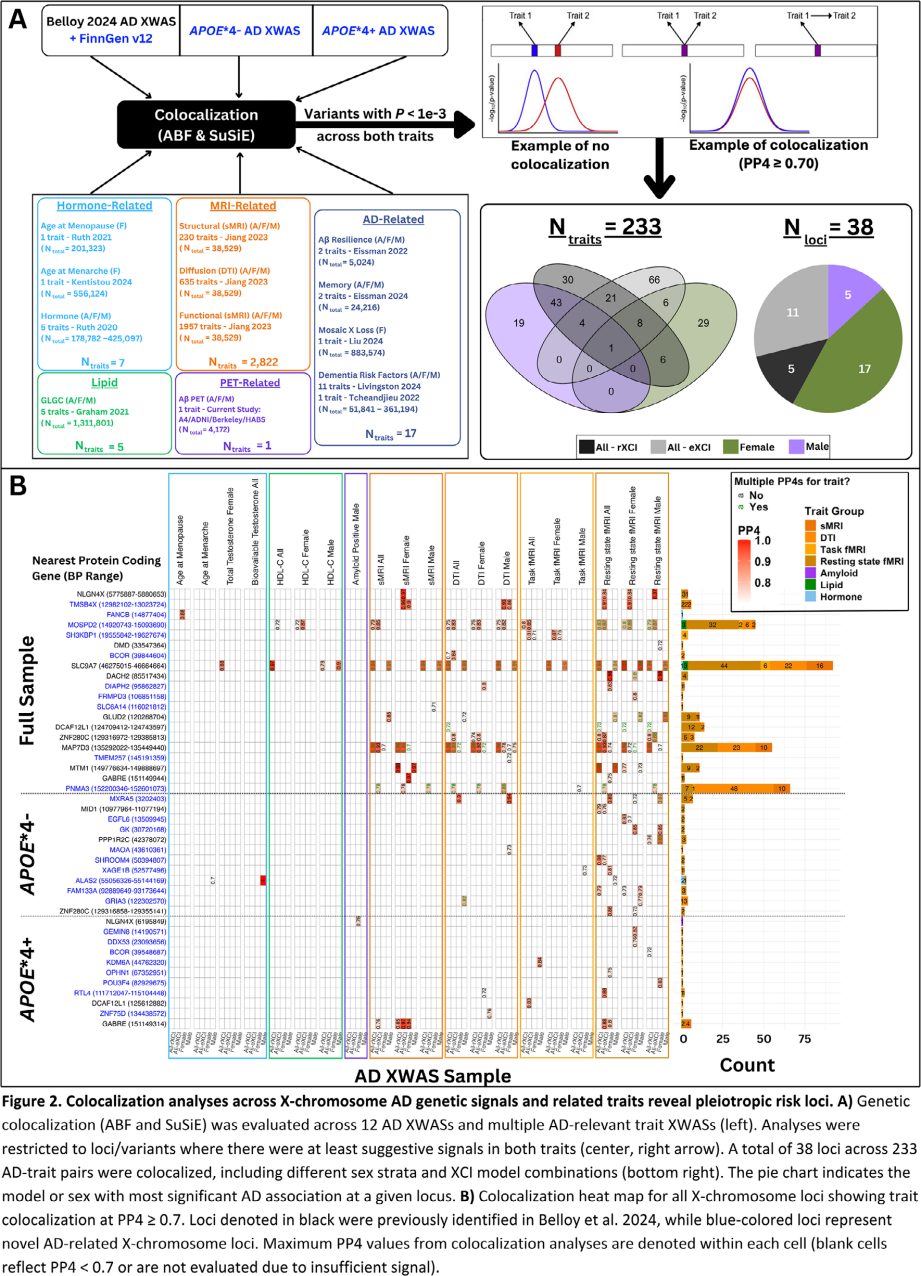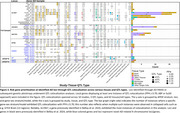# X Chromosome Genetic Risk Across Sex, APOE*4, and Pleiotropic Traits Reveals Novel Alzheimer's Disease Mechanisms and Risk Genes

**DOI:** 10.1002/alz70855_098765

**Published:** 2025-12-23

**Authors:** Noah Cook, Chenyu Yang, Danielle M. Reid, Ting‐Chen Wang, Yann Le Guen, Karly Cody, Richard Sherva, Rui Zhang, Victoria C. Merritt, Matthew S. Panizzon, Richard L. Hauger, J. Michael Gaziano, Mary Ellen I. Koran, Beth Mormino, Logan Dumitrescu, Derek Archer, Timothy J. Hohman, Mark W. Logue, Valerio Napolioni, Michael D. Greicius, Michael E. Belloy, the VA Million Veteran Program, FinnGen

**Affiliations:** ^1^ NeuroGenomics and Informatics Center, Washington University School of Medicine, St Louis, MO, USA; ^2^ Department of Neurology, Washington University School of Medicine, St Louis, MO, USA; ^3^ Vanderbilt Genetics Institute, Vanderbilt University Medical Center, Nashville, TN, USA; ^4^ Vanderbilt Memory & Alzheimer's Center, Vanderbilt University Medical Center, Nashville, TN, USA; ^5^ Department of Neurology and Neurological Sciences, Stanford University School of Medicine, Stanford, CA, USA; ^6^ Quantitative Sciences Unit, Department of Medicine, Stanford University School of Medicine, Stanford, CA, USA; ^7^ Biomedical Genetics, Boston University Chobanian & Avedisian School of Medicine, Boston, MA, USA; ^8^ National Center for PTSD, VA Boston Healthcare System, Boston, MA, USA; ^9^ Center of Excellence for Stress and Mental Health, VA San Diego Healthcare System, San Diego, CA, USA; ^10^ Department of Psychiatry, University of California San Diego, La Jolla, CA, USA; ^11^ Center for Behavior Genetics of Aging, University of California, San Diego, La Jolla, CA, USA; ^12^ University of California San Diego, La Jolla, CA, USA; ^13^ Million Veteran Program (MVP) Coordinating Center, VA Boston Healthcare System, Boston, MA, USA; ^14^ Division of Aging, Brigham & Women's Hospital, Harvard Medical School, Boston, MA, USA; ^15^ Department of Radiology, Mayo Clinic, Phoenix, AZ, USA; ^16^ Vanderbilt Memory and Alzheimer's Center, Vanderbilt University Medical Center, Nashville, TN, USA; ^17^ Department of Pharmacology, Vanderbilt University School of Medicine, Nashville, TN, USA; ^18^ Biomedical Genetics, Department of Medicine, Boston University Medical School, Boston, MA, USA; ^19^ Department of Psychiatry, Boston University Chobanian & Avedisian School of Medicine, Boston, MA, USA; ^20^ Department of Biostatistics, Boston University School of Public Health, Boston, MA, USA; ^21^ School of Biosciences and Veterinary Medicine, University of Camerino, Camerino, Macerata, Italy

## Abstract

**Background:**

Our group recently performed the largest‐to‐date X‐chromosome wide association study (XWAS) of Alzheimer's disease (AD). Here, we expand on this with additional samples, novel sex‐ and APOE*4‐stratified analyses, and evaluate escape from random X chromosome inactivation (eXCI) to better capture female‐biased risk genes. We then integrate these results with XWASs of various AD‐relevant traits to elucidate shared genetic signals and ultimately uncover novel AD risk genes and mechanisms.

**Method:**

The AD XWAS design is shown in Figure 1A and includes random X chromosome inactivation (rXCI) and eXCI models. We further assessed suggestive AD signals (*p* <1x10^‐3^) for pleiotropy using genetic colocalization with 2852 AD‐relevant traits (Figure 2A). Loci exhibiting X chromosome‐wide significance (*p* <1x10^‐5^) or apparent trait colocalization underwent quantitative trait locus (QTL) colocalization analyses across 62 tissues and cell types to functionally prioritize AD risk genes.

**Result:**

Full sample analyses reproduced Belloy et al. 2024 findings and identified 3 additional loci, including a male‐specific signal near *GLUD2* that impacts glutamate/glutamine metabolism (Figure 1B). APOE*4‐stratified analyses revealed 9 additional loci, including 3 eXCI hits in APOE*4 carriers (Figure 1C‐D). Pleiotropy colocalization identified 38 loci, most being novel and many driven by eXCI and/or female‐specific AD XWAS, including *MOSPD2, PNMA3* and *GABRE* loci that showed extensive colocalization with brain imaging traits (Figure 2B). The most significant XWAS locus, *SLC9A7*, colocalized with total testosterone, HDL‐C, and various brain imaging traits. Only the *NLGN4X* locus colocalized with amyloid positivity, linking its proposed role in synaptic dysfunction to amyloid pathology. The locus at *KDM6A*, previously implicated as an AD resilience gene, colocalized with a functional MRI (fMRI) phenotype. QTL colocalization analyses confirmed gene prioritizations in Belloy et al. 2024, and additionally provided support for *PNMA3*, an antigen highly expressed in the brain, and 3 *APOE*‐stratified loci, including *ACLS4*, which impacts lipid metabolism.

**Conclusion:**

This study provides novel insights into biological mechanisms that contribute to X‐linked genetic risk for AD in an APOE*4 and sex‐specific manner, identifying various novel risk loci. Notably, 29 female‐biased risk loci were uncovered, which may shed light on sex disparities in AD. Overall, our findings will aid in elucidating AD pathways and drug targets relevant to personalized medicine.